# Protein Recognition and Amyloid Remodeling Governed by Paddlewheel Diruthenium Coordination Chemistry

**DOI:** 10.1021/acs.inorgchem.6c02947

**Published:** 2026-08-19

**Authors:** Sara La Manna, Daniele Florio, Giarita Ferraro, Maria Rosaria Policino, Filomena Altieri, Aarón Terán, Santiago Herrero, Antonello Merlino, Daniela Marasco

**Affiliations:** † Department of Pharmacy, University of Naples Federico II, 80131 Naples, Italy; ‡ 591458 IRCCS SYNLAB SDN, Via G. Ferraris 144, Naples 80146, Italy; § Department of Chemical Sciences, University of Naples Federico II, 80126 Naples, Italy; ∥ Structural Biology Programme, Spanish National Cancer Research Centre (CNIO), Melchor Fernández Almagro 3, 28029 Madrid, Spain; ⊥ MatMoPol Research Group, Department of Inorganic Chemistry, Faculty of Chemical Sciences, Complutense University of Madrid, Avenida Complutense s/n, 28040 Madrid, Spain; # Knowledge Technology Institute, Complutense University of Madrid, Campus de Somosaguas, 28223 Pozuelo de Alarcón, Madrid, Spain

## Abstract

Paddlewheel diruthenium (Ru_2_) complexes are promising modulators of protein aggregation due to their tunable coordination chemistry and dual-action properties. Here, we investigate the interaction of five Ru_2_ complexes with hen egg white lysozyme (HEWL), an amyloid model, to elucidate their antiaggregation capabilities. High-resolution X-ray crystallography shows that all complexes preferentially bind to Asp119 and, in some cases, Asp101, through coordination to the Ru_2_ core while preserving the overall protein fold. Both covalent and noncovalent interactions are observed, depending on ligand environment and steric effects. Solution studies confirm the formation of HEWL–Ru_2_ adducts under both neutral and acidic conditions. Functional assays demonstrate that all Ru_2_ complexes effectively inhibit HEWL fibrillogenesis, as indicated by reduced ThT fluorescence and the absence of large aggregates in dynamic light scattering measurements. Disaggregation of preformed fibrils was more variable, with the complex bearing vacant axial sites showing the highest activity. Circular dichroism and scanning electron microscopy analyses reveal that these compounds redirect aggregation toward noncanonical morphologies rather than fully dissolving fibrils. Cytotoxicity assays confirm reduced HEWL-induced cellular toxicity. Overall, our findings establish a correlation between ligand composition, coordination behavior, protein binding, and antiamyloid activity, providing a framework for designing Ru_2_-based multifunctional modulators of protein aggregation.

## Graphical abstract

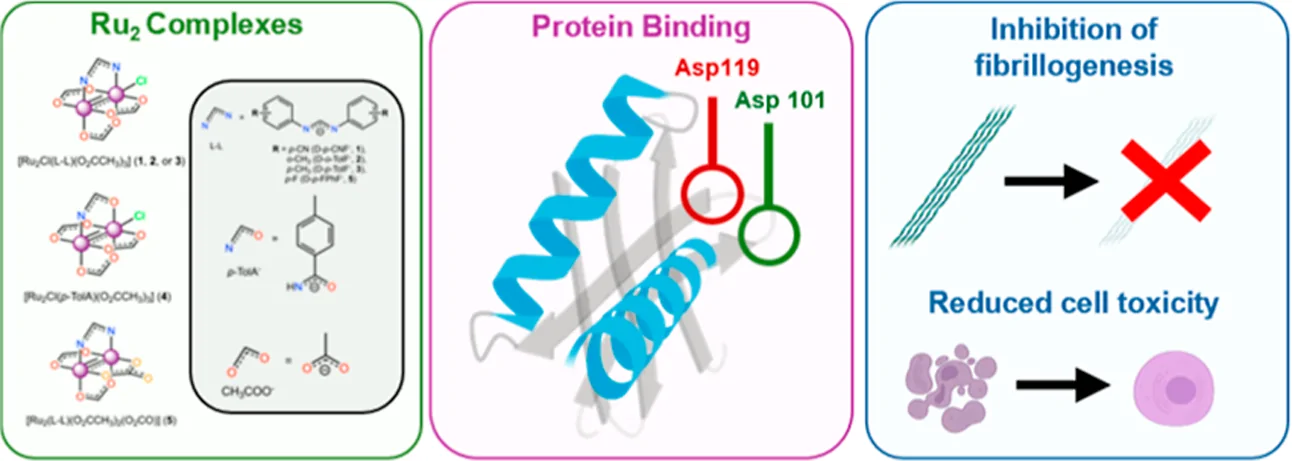

## Introduction

Hen egg white lysozyme (HEWL) is a small (∼14 kDa) globular enzyme extensively used as a model protein to study amyloid fibril formation, aggregation mechanisms, and screening of antiamyloid compounds. Its popularity stems from its well-characterized structure, commercial availability, and reliable conversion into β sheet-rich fibrils under controlled denaturing conditions, which mimic key features of pathogenic amyloids observed in human diseases such as Alzheimer’s and systemic amyloidosis.^1^ Common experimental conditions, acidic pH, elevated temperature, chemical denaturants, or agitation, induce partial unfolding and nucleation of HEWL into amyloid fibrils, which can be monitored by spectroscopic and microscopic techniques. Although HEWL itself is not a major pathological amyloid in humans, its structural homology (∼60% sequence identity) to human lysozyme, mutations in which cause hereditary systemic amyloidosis,^2^ makes it a biologically relevant surrogate for probing amyloidogenesis principles and the molecular determinants of fibril formation.^3^ HEWL aggregation follows a nucleation-dependent polymerization mechanism, proceeding through partially unfolded intermediates that self-assemble into oligomers and eventually mature fibrils with a characteristic cross-β architecture.^4^ The robustness and reproducibility of HEWL fibrillation make it an ideal platform for screening amyloid aggregation inhibitors and disaggregating agents.^5^ Indeed, beyond inhibition, HEWL is also a valuable model for studying amyloid disaggregation. Chemical chaperones, polysaccharides, and nanomaterials can remodel preformed fibrils and promote partial refolding, providing mechanistic insight into fibril stability and reversibility.^6^ Both natural and synthetic compounds have demonstrated efficacy in modulating HEWL fibrillogenesis. For instance, the triterpene glycyrrhizic acid inhibits HEWL fibril formation by binding aggregation-prone regions and redirecting partially unfolded monomers toward soluble species.^7^ Synthetic 4-methylthiocoumarin derivatives exhibit dual activity by suppressing both fibril formation and disassembling preformed fibrils by targeting both nucleation and fibril stabilization.^8^ Other small molecules, such as 1,3,5-triphenylbenzene derivatives, inhibit fibril growth in a substituent-dependent manner, with electron-withdrawing groups enhancing interactions with amyloidogenic intermediates and reducing associated cytotoxicity.^9^ Among natural compounds, polyphenols, including rosmarinic acid and resveratrol, not only inhibit fibrillogenesis but also destabilize preformed fibrils, providing cytoprotective effects by modulating protofibril stability.^10^ Flavonoids such as taxifolin redirect aggregation toward non-β-sheet globular aggregates, suppressing typical fibril assembly and reducing toxicity.^11^ Searching for aggregation modulators, coordination compounds offer a tunability that small organic molecules often lack. By independently modifying the ligand properties—including electronic properties, steric effects, and solubility—as well as the identity and number of the metal centers, metal complexes can be tailored to selectively bind fibrillar surfaces or early oligomeric intermediates, thereby acting as inhibitors of nucleation, elongation blockers, or fibril disruptors.^12–15^ Furthermore, some complexes exhibit fluorescent or luminescent properties, enabling simultaneous tracking of aggregation and disaggregation events in real time, a feature highly valuable for mechanistic studies.^12^ Integrating metal-based strategies with conventional small-molecule inhibitors could provide synergistic approaches to prevent or reverse amyloid pathology in protein misfolding diseases.

Paddlewheel diruthenium (Ru_2_) complexes have emerged as a novel class of metallodrugs with intriguing therapeutic potential rooted in their bimetallic Ru–Ru core characterized by an Ru­(II)–Ru­(III) mixed-valence state and a metal–metal bond order of 2.5, a tunable ligand environment (comprising both equatorial and axial positions), and the ability to interact directly with biological targets.^16–21^ The distinctive paddlewheel architecture offers a versatile platform for drug design. For example, bioactive molecules have been coordinated at the equatorial positions of the Ru_2_ core,^22–26^ enabling the construction of dual-action prodrugs in which the bimetallic core acts synergistically with the released therapeutics—a feature that positions these complexes as so-called dual-action metallodrugs in modern medicinal chemistry.^20,24^ Beyond the established anticancer activity of these compounds,^18,19,24^ paddlewheel Ru_2_ complexes have recently been shown to reduce amyloid fiber formation of Aβ_1–42_ and abrogate peptide cytotoxicity in vitro, pointing to potential neurotherapeutic applications.^27,28^ Understanding how these compounds interact with proteins at the molecular level has proven essential to rationalize their antiaggregation activity and to establish design principles for next-generation metallodrugs. Our groups have obtained high-resolution X-ray structures of different Ru_2_/protein adducts under multiple crystallization conditions.^29–34^ Early studies conducted to understand the protein-binding properties of Ru_2_ complexes used the common starting material [Ru_2_Cl­(O_2_CCH_3_)_4_].^30^ However, this species exhibits limited stability in solution and tends to aggregate over time. The substitution of one or two of the acetate ligands by formamidinate (L–L) ligands in this derivative gave rise to [Ru_2_Cl­(L–L)­(O_2_CCH_3_)_3_] and [Ru_2_Cl­(L–L)_2_(O_2_CCH_3_)_2_] complexes with higher stability in various aqueous media.^31,33^ To date, the investigated Ru_2_ complexes were characterized by the presence of acetate, formamidinate, and/or carbonate ligands coordinated at the equatorial positions of the Ru_2_ core. In this article, we studied the effect of five Ru_2_ complexes (Figure 1) on the fibrillogenic properties of HEWL. We investigated for the first time how an amidate (*p*-TolA) ligand at the equatorial position (complex **4**) affects the protein binding properties of Ru_2_ compounds and how this might influence the antiaggregatory properties of these species. Furthermore, we examine the role of the axial coordination sites in modulating the antiaggregation properties of diruthenium complexes: compounds **1**–**4** bear an axial chloride ligand, whereas complex **5** features both axial positions vacant. In this work, we demonstrate that the coordination environment of paddlewheel Ru_2_ complexes directly controls their protein-binding properties and antiaggregation behavior, providing design principles for the development of coordination-driven modulators of amyloid assembly.

**Figure 1:**
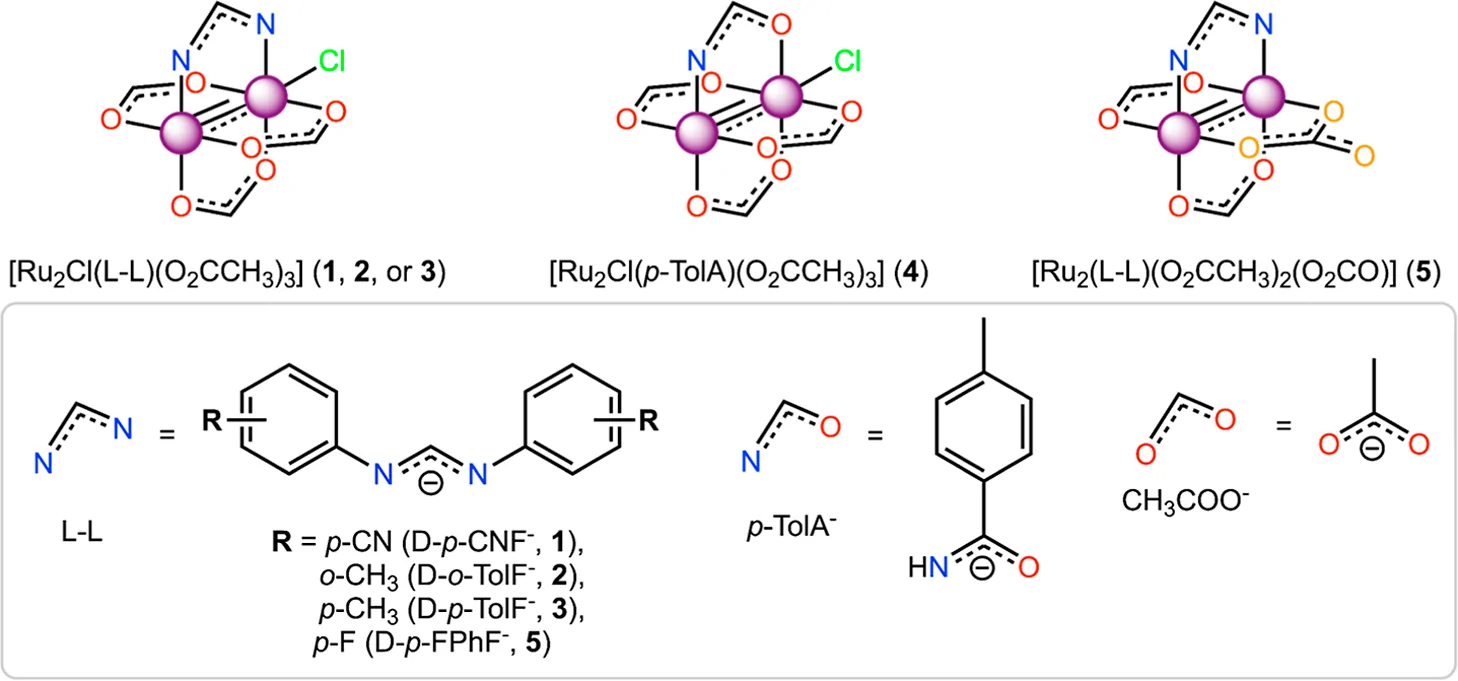
Schematic description of Ru_2_ compounds **1**–**5** investigated in this study.

## Results and Discussion

### X-ray Structural Analysis of the Adducts Formed by HEWL with the Ru_2_ Compounds

To understand the protein binding properties of compounds **1**–**5** with HEWL, X-ray crystallography was used. The 3D structure of the adducts formed by each complex with HEWL was solved (Figure 2). Crystals of the adducts were prepared by the soaking method (see Experimental Section). These crystals diffracted X-rays at a resolution within the range of 1.13–1.75 Å. Data collection and refinement statistics are reported in Table S1. The structures were deposited in the PDB (https://www.rcsb.org) as entries 29LJ (HEWL/**1**), 29MY (HEWL/**2**), 29NX (HEWL/**3**), 29MN (HEWL/**4**), and 29MR (HEWL/**5**).

**Figure 2:**
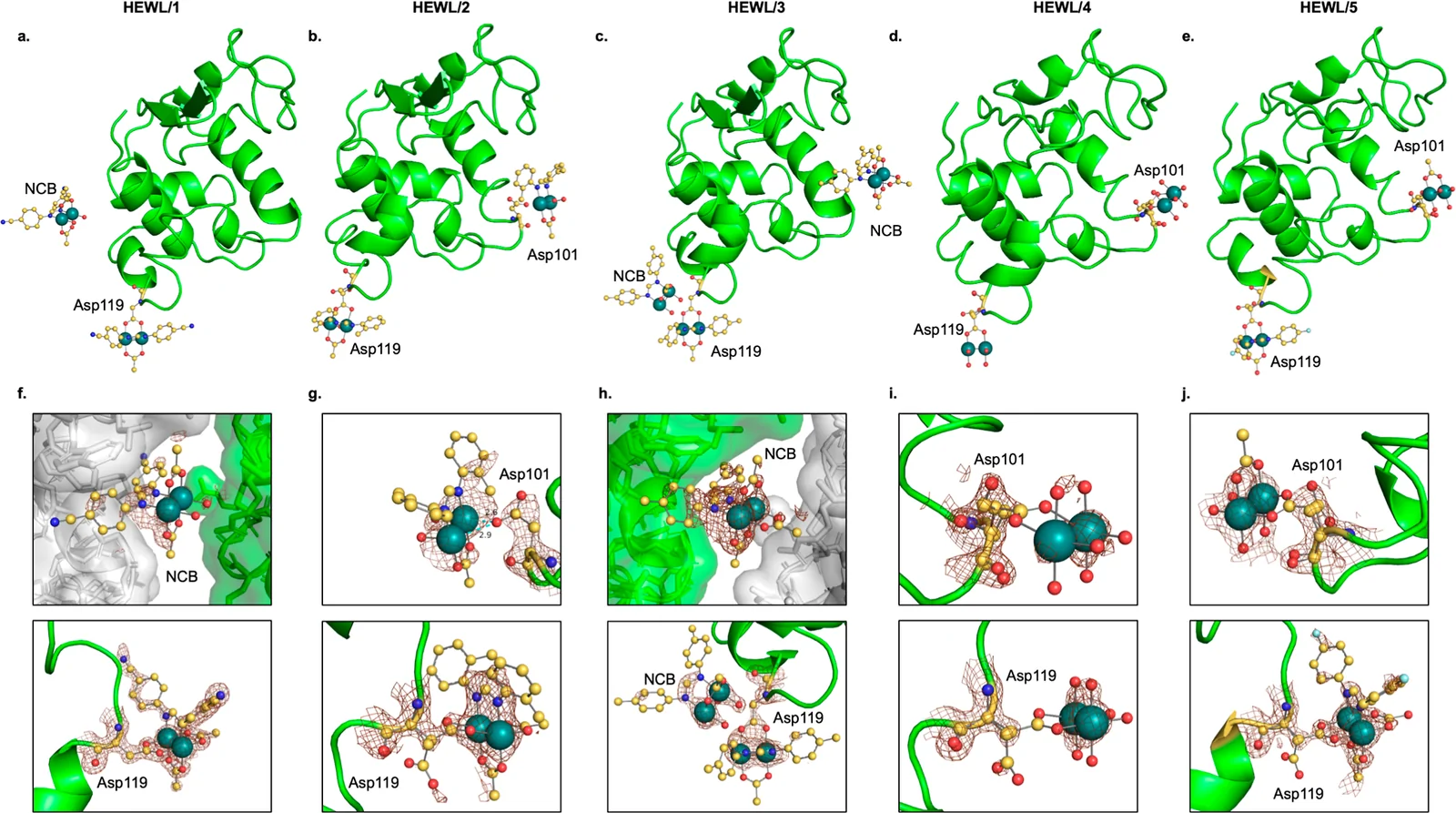
(**a**–**e**) Overall structures of the adducts formed by complexes **1**–**5** with HEWL in the solid state. (**f**–**j**) Close-up view of the diruthenium binding sites in the formed adducts. 2*F*o–*F*c electron density map is contoured at 1σ level (in brown). Symmetry-related HEWL molecules are colored in gray. Atom color code for the Ru_2_ complexes is yellow for C, blue for N, red for O, turquoise for Ru, and light blue for F. NCB indicates noncovalently bound Ru_2_ fragments.

The overall structure of the adducts formed by HEWL with complexes **1**–**5** is nearly identical to that of the metal-free protein (Figure 2a–e). The root-mean-square deviation (rmsd) of the carbon alpha atoms between the metal-free protein and the Ru_2_/HEWL adducts is within the range 0.11–0.15 Å. The main differences are located at the level of the Ru_2_ binding sites (Figure 2f–j). Previously, we described that charge affects the protein binding properties of diruthenium derivatives.^31,32,34^ Anionic complexes tend to remain on the protein surface bound to protein atoms through noncovalent interactions, unless equatorial ligand substitution occurs,^32^ while cationic complexes are coordinated by donors of the side chains of Asp, Lys, His, Arg, and Glu, through their axial or equatorial positions.^33^

In all the structures of the adducts here solved, the side chain of Asp119 is the preferential binding site: one acetate ligand of the Ru_2_ complex is replaced by the carboxylate group of the Asp side chain. Another binding site is observed close to the side chain of Asp101 in the adducts with compounds **2**, **4**, and **5**. Notably, in the adducts with **1** (Figure 2a and f) and **3** (Figure 2c and h), noncovalently bonded fragments (NCB) were found. This is the first time we observe that cationic complexes interact with a protein via noncovalent interactions. This type of bond could be favored by the presence of steric substituents on the aromatic rings of **1** and **3**.

In general, the Asp side chain coordinates to the bimetallic core through either axial or equatorial sites, preserving the characteristic paddlewheel geometry. However, the HEWL/**2** adduct reveals an unusual binding mode at Asp101 (Figure 2b and g). Here, only one oxygen atom of the carboxylate group bridges the two ruthenium centers, with Ru–O distances of 2.6 and 2.9 Å. These elongated distances suggest a weak, asymmetric interaction, reminiscent of previously reported entatic states in diruthenium metalloproteins—transient species that are stabilized only within the protein scaffold and would otherwise be unstable in solution.^29^ The present observation may represent another example of such a protein-trapped intermediate. This new form of interaction can be associated with the steric hindrance of the methyl group at the *ortho* position in the aromatic ring.

In the structure of HEWL/**4** (Figure 2d and i), the presence of the amidate ligand in the starting compound does not affect the protein-binding properties of the complex, yielding a result similar to that observed in the case of the formamidinate analogues. Nevertheless, we were unable to clearly identify the electron density of the amidate ligand, probably due to the high mobility of the phenyl ring. For this reason, we assigned both positions as [Ru_2_(Asp)­(OH_2_)_6_]^4+^ species.

### Adduct Formation of HEWL with Ru_2_ Compounds (UV–vis Absorption Spectroscopy and Electrospray Mass Spectrometry Analysis)

The stability of related Ru_2_ compounds, such as [Ru_2_Cl­(L–L)­(O_2_CCH_3_)_3_] and [Ru_2_Cl­(L–L)_2_(O_2_CCH_3_)_2_] (L–L = formamidinate ligands), has previously been established in various aqueous media, which were then used as crystallization conditions (from pH = 4 to pH = 7.5).^27,28,30,33,34^ However, to form HEWL amyloid fibrils, a highly acidic pH is required (20 mM Gly–HCl at pH = 2).^3^ Consequently, the first step was to evaluate the stability of complexes **1**–**5** using UV–vis absorption spectroscopy in low pH acidic conditions (Figure S1).

Although slight spectral variations were observed over the 8 day incubation period, the overall absorption profiles remained essentially unchanged, indicating that the Ru_2_ complexes are largely stable under the aggregation-inducing conditions. These minor changes are likely associated with slight modifications of the coordination environment under acidic conditions rather than with significant decomposition of the complexes.

Then, the formation of adducts in solution by **1**–**5** with HEWL was analyzed by electrospray ionization mass spectrometry (ESI-MS) under both neutral and acidic conditions. The spectra for HEWL/**1** and HEWL/**2**–**5** are shown in Figures 3 and S2, respectively, with peak assignments summarized in Tables S2 and S3. In all cases, a similar behavior was observed: HEWL forms adducts with Ru_2_-containing fragments derived from the starting Ru_2_ complex following the loss of one acetate, one chloride, and one water ligand. This in-solution behavior corroborates crystallographic observations identifying the axial chloride ligand as labile and readily replaceable by water. Moreover, the loss of the acetate ligand is necessary in the Ru_2_ complexes to coordinate to aspartate side chains in positions 101 and 119. It seems that despite the reduction of pH from the crystallographic (pH = 4) to reactivity (pH = 2) studies, all the evidence suggests that the diruthenium species continue to behave in a similar manner, with the binding of Ru_2_ complexes **1**–**5** to the HEWL structure after acetate release.

**Figure 3:**
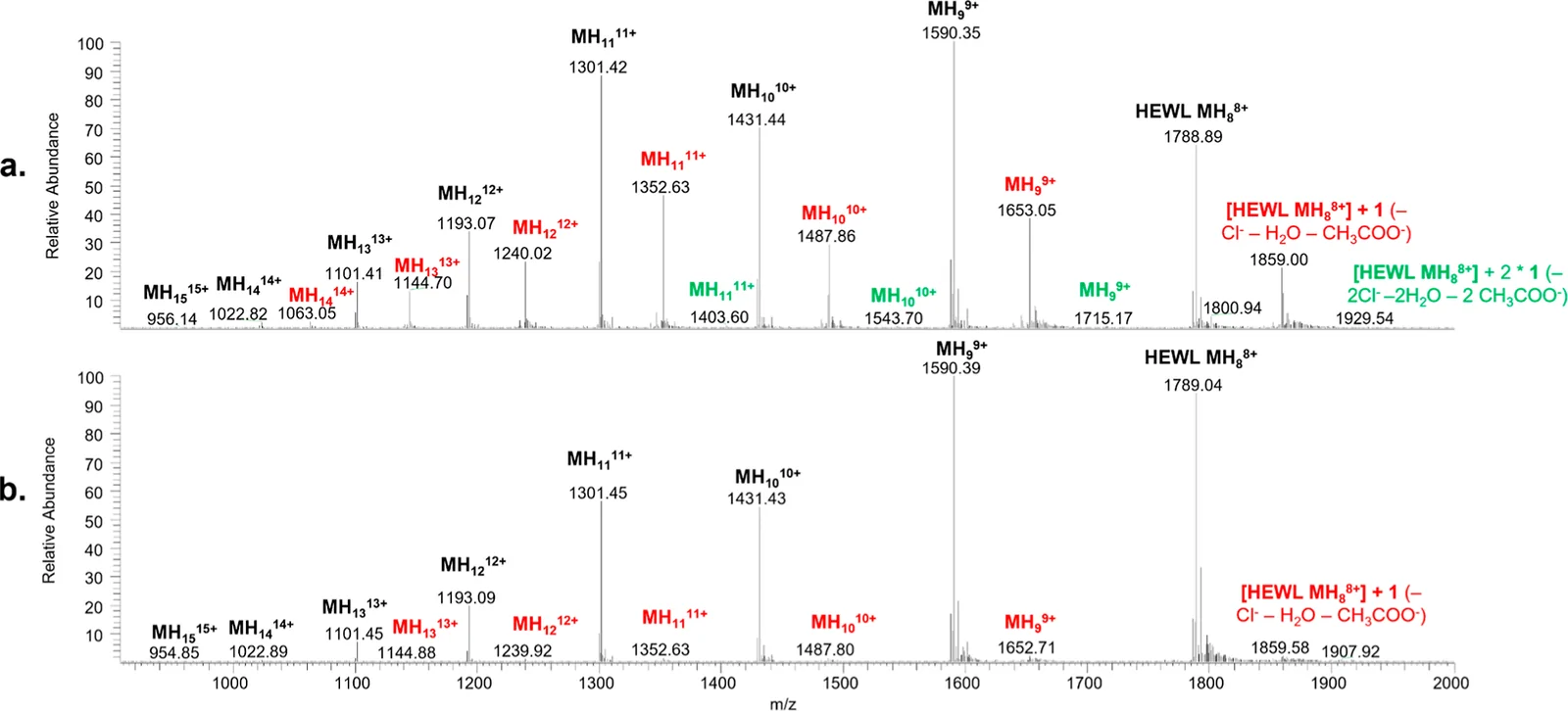
ESI-MS spectra of the HEWL after incubation for 24 h with complex **1** in water (a) and in 20 mM Gly-HCl buffer at pH = 2 (b) at a 1:1 HEWL/**1** molar ratio. Signals in black correspond to fragmentation of HEWL alone, while signals in red and green correspond to two distinct adducts formed with complex **1**.

### Inhibition and Remodeling of HEWL Aggregates by Ru_2_ Compounds

To assess the inhibitory effect of Ru_2_ complexes on HEWL aggregation, we performed thioflavin T (ThT) fluorescence assays. ThT is a benzothiazole dye that exhibits weak fluorescence in aqueous solution because excited-state energy is dissipated through intramolecular rotation. Upon binding to the ordered cross-β structure of amyloid fibrils, these rotational motions are restricted, suppressing nonradiative decay and resulting in a marked enhancement of fluorescence emission. Therefore, the fluorescence intensity is proportional to the amount of amyloid fibrils present.^35^

Here, we evaluated the performance of complexes **1**–**5** in two modes: inhibition and disaggregation. In the inhibition assay, Ru_2_ compounds were preincubated with the protein at the beginning of the aggregation process. In the disaggregation assay, Ru_2_ compounds **1**–**5** were added after HEWL aggregation occurred (168 h according to the DLS analysis, see below).

In the inhibition assay (Figure 4a), the ThT fluorescence measurements revealed that HEWL undergoes a pronounced time-dependent increase in amyloid formation under the assay conditions, whereas coincubation with Ru_2_ complexes significantly attenuates this process. Notably, complexes **1**, **2**, and **5** strongly suppress ThT fluorescence throughout the incubation period, while compounds **3** and **4** permit a moderate fluorescence increase at later time points, suggesting partial effects in altering aggregation pathways. On the other hand, in the disaggregation assay (Figure 4b), the incubation of preformed HEWL aggregates with the same compounds resulted in a time-dependent partial reduction of ThT signals for all samples except for **4**, albeit with different rates and final fluorescence levels: compound **5** was the most active in the disaggregation. This could be explained by the different coordination environment between complexes **1**–**4** and compound **5**. In the case of compounds **1**–**4**, in the solid state all molecules have a chloride ligand bonded to the axial position, which can bridge two diruthenium units, giving rise to a polymeric structure, as reported in the crystal structures of complexes **1** and **4**.^36^ However, in the case of complex **5**, all coordinated ligands are at the equatorial positions, leaving the axial sites vacant. Axial ligands seem to reduce the reaction kinetics, as they must first be released before binding, giving rise to lower reactivity in compounds **1**–**4** compared to complex **5**. The enhanced activity observed for compound **5** highlights the critical role of axial-site lability in regulating the interaction kinetics of paddlewheel diruthenium complexes with aggregated protein species.

**Figure 4:**
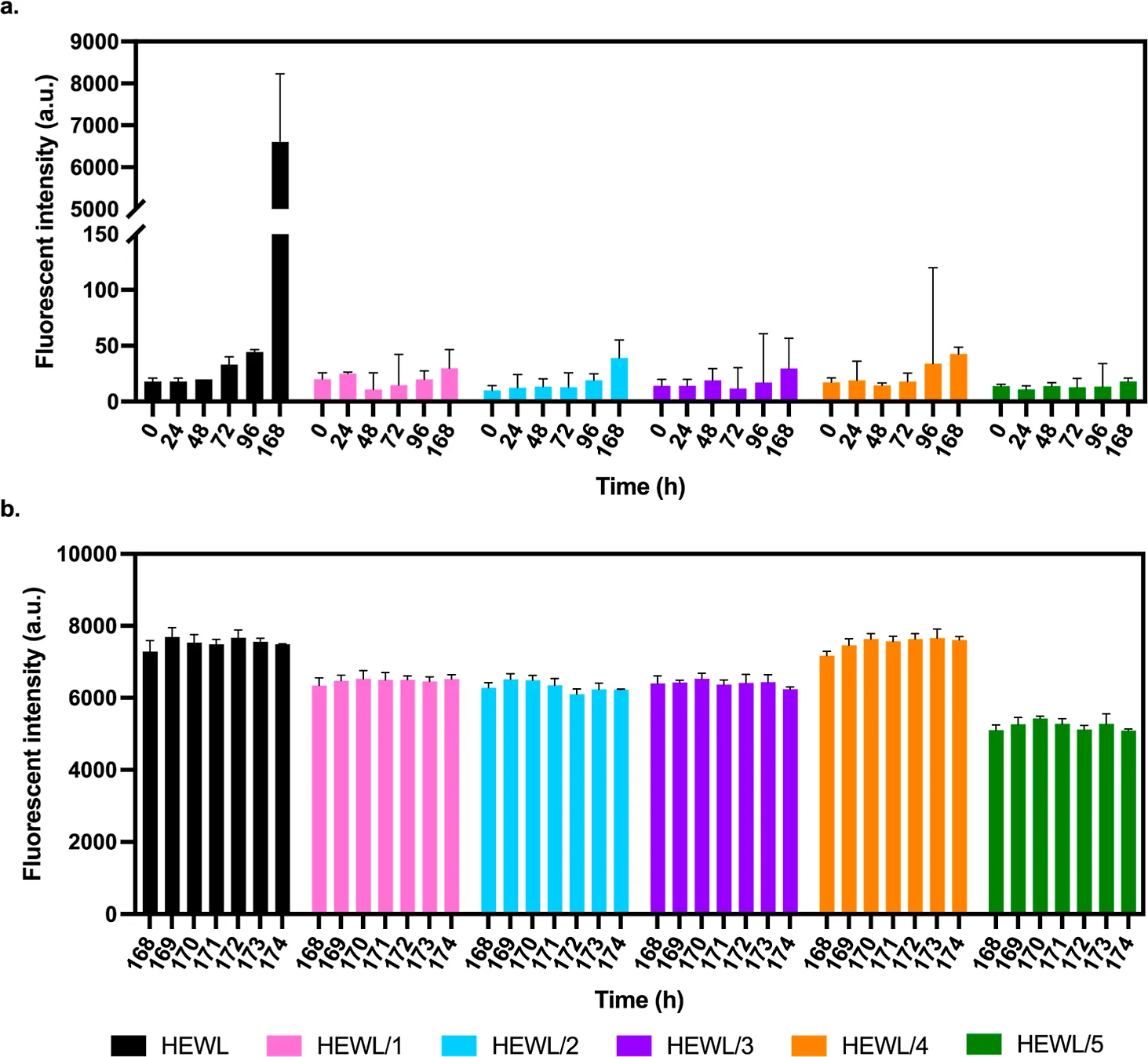
Inhibition (a) and disaggregation (b) of amyloid aggregation of HEWL monitored by ThT fluorescence in the absence and presence of Ru_2_ complexes. HEWL (50 μM) was incubated in 20 mM Gly–HCl buffer (pH 2.0) in the absence or presence of Ru_2_ complexes **1**–**5** at a 1:1 HEWL/Ru_2_ complex molar ratio. Fluorescence measurements were performed at 25 °C using excitation and emission wavelengths of 440 and 483 nm, respectively. Fluorescence intensity is reported in arbitrary units (a.u.). Note the broken *y*-axis, introduced to accommodate the large difference in fluorescence intensity between HEWL alone and treated samples. Average of three independent experiments.

Notably, the inhibitory potency of Ru_2_ compounds does not directly translate to enhanced disaggregation efficiency, suggesting that while all Ru_2_ complexes interfere with HEWL aggregation, their abilities to destabilize or remodel preformed aggregates differ, supporting the notion that aggregation inhibition and fibril disassembly involve related but not identical mechanisms.

### Aggregate Size Reduction by Ru_2_ Complexes

Dynamic light scattering (DLS) analysis provided complementary information on the aggregation state of HEWL in both inhibition and disaggregation experiments. Right correlation experiments are reported in Figure 5. Metal-free HEWL (Figure 5a and b) displayed a predominant population of large aggregates, with intensity-weighted size distributions centered in the hundreds of nanometers range and a relatively high polydispersity index, consistent with fibrillar aggregation.^37^ Notably, samples of HEWL incubated with Ru_2_ complexes did not show measurable correlation functions in the investigated time range (0–168 h), indicating the absence of large scattering particles and, therefore, effective inhibition of aggregate formation at a 1:1 HEWL/Ru_2_ complex molar ratio, for all compounds. Conversely, the addition of Ru_2_ compounds to preformed HEWL aggregates (Figure 5c and d) resulted in detectable changes in the hydrodynamic size distributions (Table S4): HEWL alone displayed a Z-average hydrodynamic diameter of 219.4 nm with a PDI of 0.337, indicative of large and heterogeneous aggregates; the addition of the Ru_2_ complexes caused a decrease of the average particle dimension, indicating a reduction in aggregate size; in particular, **5** provided the strongest effect with a diameter of 151.9 nm and a modest decrease in the PDI value along with **1**, suggesting a partial reduction in aggregate heterogeneity. Despite observed small changes, large aggregate populations were still detected in the presence of Ru_2_ complexes, indicating that they do not completely disassemble the fibrillar structures but rather promote partial remodeling or fragmentation of the aggregates.

**Figure 5:**
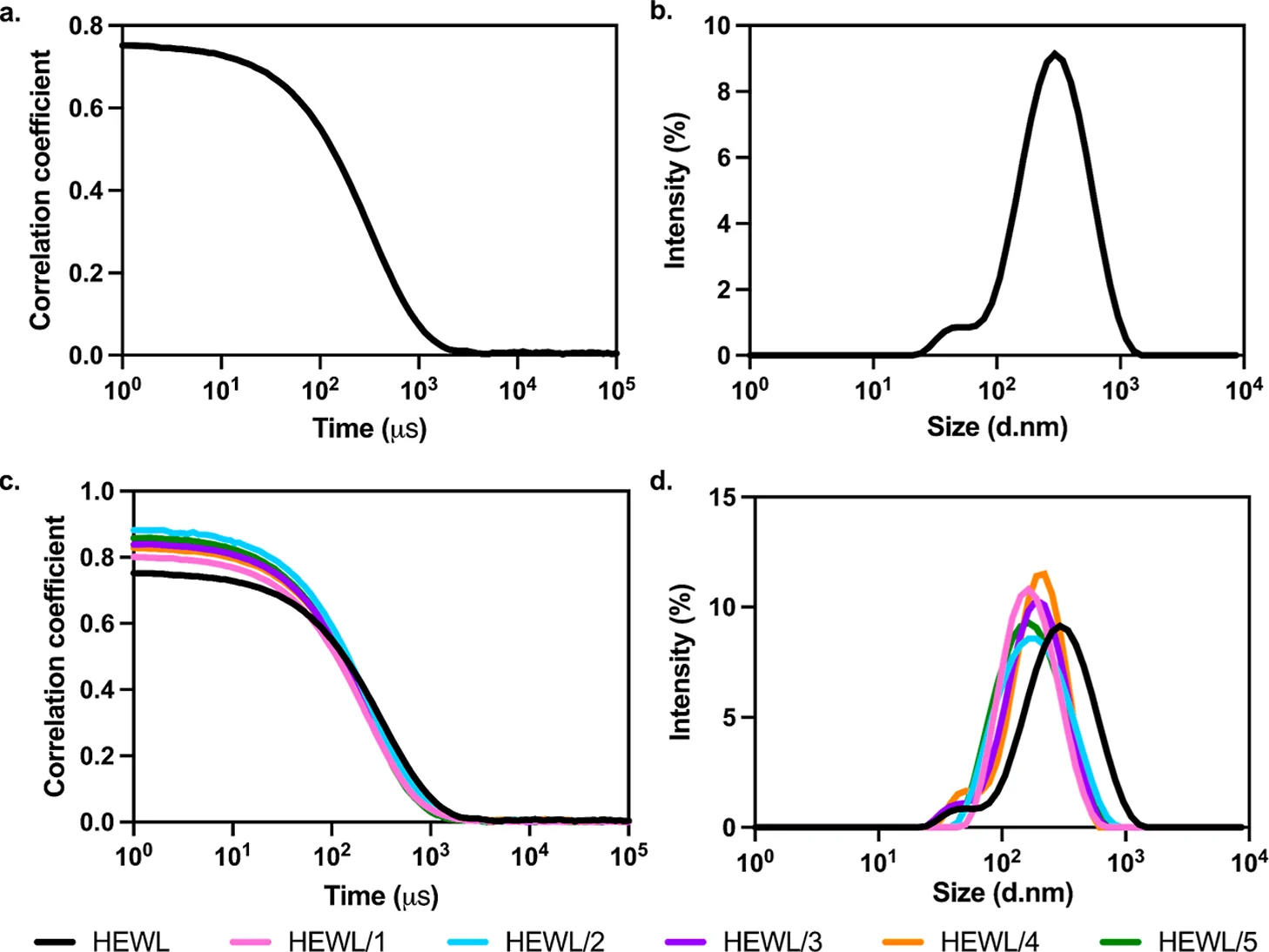
DLS analysis of HEWL aggregation in inhibition **(a-b)** and disaggregation **(c**–**d)** experiments. DLS correlation functions **(a, c)** and intensity-weighted size distributions **(b, d)** of HEWL (50 μM) incubated in 20 mM Gly–HCl buffer (pH 2.0) at 25 °C in the absence or presence of Ru_2_ compounds **1**–**5** at a 1:1 HEWL/Ru_2_ complex molar ratio. Panel (**a, b**) shows the sample analyzed after 168 h of incubation under inhibition conditions, whereas panel (**c, d**) shows samples analyzed under disaggregation conditions.

### Secondary Structure Changes Induced by Ru_2_ Compounds

We used far-UV circular dichroism (CD) spectroscopy to detect alterations in the HEWL secondary structure in the absence and presence of the Ru_2_ compounds **1**–**5** in acidic conditions at different times. HEWL is known to adopt a predominantly α-helical fold, typically characterized by negative CD bands at ∼211 and 220 nm, with comparable Cotton effects.^38^ Consistent with this structural signature, the spectrum of HEWL alone at *t* = 0 h and pH = 2 displays pronounced negative bands in the 210–225 nm region, indicative of the preserved native conformation (Figure 6, left panel, black line). Upon the addition of the Ru_2_ compounds, the spectra exhibit detectable differences in ellipticity and band shape, suggesting that binding of the compounds induces early perturbations in the local protein secondary structure environment (Figure 6, left panel). In detail, the presence of Ru_2_ compounds determined a shift of the absolute minimum from 220 nm to ∼227 nm with an enhanced CD signal and a shoulder at ∼216 nm. These CD profiles are indicative of a greater β-sheet content when compared to metal-free HEWL.

**Figure 6:**
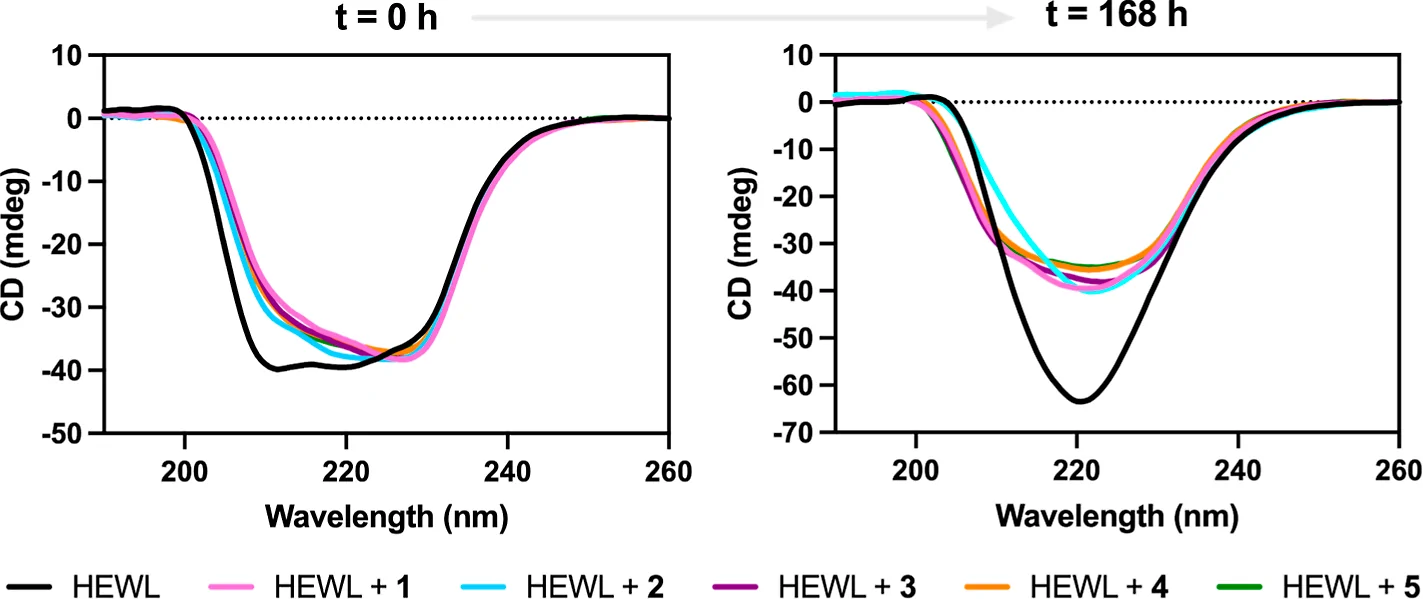
Far-UV circular dichroism (CD) spectra of HEWL (50 μM) in 20 mM Gly–HCl buffer (pH 2.0) recorded at 25 °C, in the absence and presence of Ru_2_ compounds **1**–**5** at a 1:1 HEWL/Ru_2_ complex molar ratio. Spectra were recorded at *t* = 0 h **(left)** and *t* = 168 h **(right)**.

After 168 h of aggregation, HEWL underwent an α→β structural transition, forming β-sheet-rich amyloid aggregates with enhanced CD signal, as largely reported (Figure 6, right panel).^39^ For most of the Ru_2_ complexes, the overall spectral shape remains similar to that observed in the case of metal-free HEWL, even though a smaller Cotton effect can be detected, which suggests how effectively Ru_2_ complexes could help the protein to maintain its secondary structure. We also consider the possible effect of Ru_2_ complexes on preformed HEWL aggregates (disaggregation mode). However, they did not dramatically alter the overall protein conformation (Figure S3), just a slight reduction in the intensity of the Cotton effect, suggesting minor structural remodeling or partial destabilization of the β-sheet content. This effect may reflect partial fibril loosening or restructuring rather than complete disassembly, consistent with the observations from DLS experiments.

### Morphological Alterations of HEWL Fibrils

The morphology of HEWL amyloid fibrils was investigated by scanning electron microscopy (SEM) to assess potential effects of Ru_2_ complexes **1**–**5** during the inhibition and disaggregation experiments (Figures 7 and S4). HEWL samples, in the absence or in the presence of Ru_2_ complexes at a 1:1 HEWL/Ru_2_ complex molar ratio, were analyzed after 7 days of incubation under aggregation-promoting conditions. Representative micrographs for HEWL/**1**, HEWL/**4**, and HEWL/**5** are shown in Figure 7b and c.

**Figure 7:**
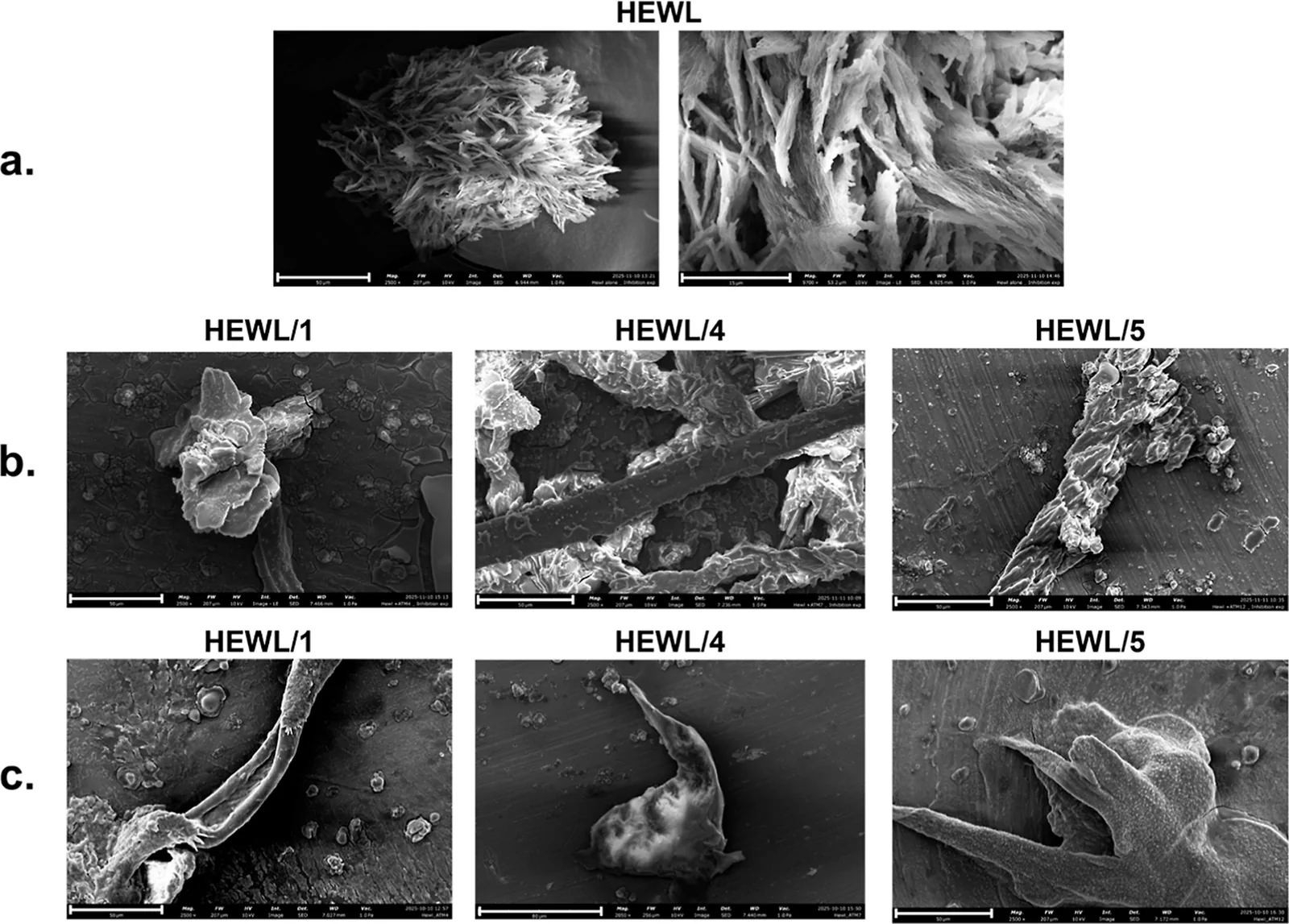
SEM micrographs of HEWL alone (a) and in the presence of Ru_2_ complexes **1**, **4,** and **5** showing representative morphological features from inhibition (b) and disaggregation (c) experiments.

SEM images of HEWL alone (Figure 7a) reveal dense fibrillar aggregates uniformly distributed across the substrate surface. These aggregates are composed of long, thin, unbranched fibrils that are highly extended, intertwining to form highly compact and interconnected networks.^40^ In the presence of Ru_2_ complexes (Figure 7), these typical fibrils are completely absent. In the inhibition assay, the Ru_2_ complexes prevent formation of the extended network, producing layered or radially clustered aggregates with occasional short and thick fibrils (Figure 7b). On the other hand, during the disaggregation assay, the preformed fibrils are fragmented into smaller, collapsed, or broken aggregates, demonstrating effective disruption of mature amyloid assemblies (Figure 7c). Although the overall morphological changes are similar across all complexes, subtle differences are observed: complexes **1** and **4** (Figure 7c) induce irregular aggregates with some short fibrils and thicker, more mature fibrillar elements, an effect also seen for complexes **2** and **3** (Figure S4), whereas complex **5** (Figure 7c) produces larger and more densely packed assemblies. A comparable trend is also observed in disaggregation, with a similar pattern of aggregate size and density changes across all Ru_2_ complexes.

### Evaluation of Cytoprotective Effects of Ru_2_ Complexes

The MTS assay was performed to evaluate the potential protective effects of Ru_2_ complexes **1**–**5** against HEWL amyloid-induced cytotoxicity (Figure 8). The intrinsic effect of Ru_2_ complexes alone on SH-SY5Y neuroblastoma cells was first evaluated, showing no significant cytotoxicity and sustained high cell viability (data not shown). As expected, treatment with preaggregated HEWL alone resulted in a measurable decrease in cell viability when compared to the control, confirming the cytotoxic nature of amyloid species.^41^ Co-incubation of HEWL with Ru_2_ complexes **1**–**5** significantly attenuated this effect, as reflected by an increase in cell viability relative to HEWL-treated cells (Figure 8). These results indicate that all Ru_2_ complexes inhibit HEWL aggregation pathways, in agreement with the in vitro aggregation assays. In contrast, in disaggregation experiments (Figure S5), the treatment of preformed HEWL aggregates with Ru_2_ complexes **1**–**5** did not lead to a significant rescue of cell viability, which remained comparable to that observed in HEWL-treated cells. This suggests that, under the present experimental conditions, the ability of Ru_2_ complexes to remodel preformed aggregates is insufficient to fully mitigate their cytotoxic effects.

**Figure 8:**
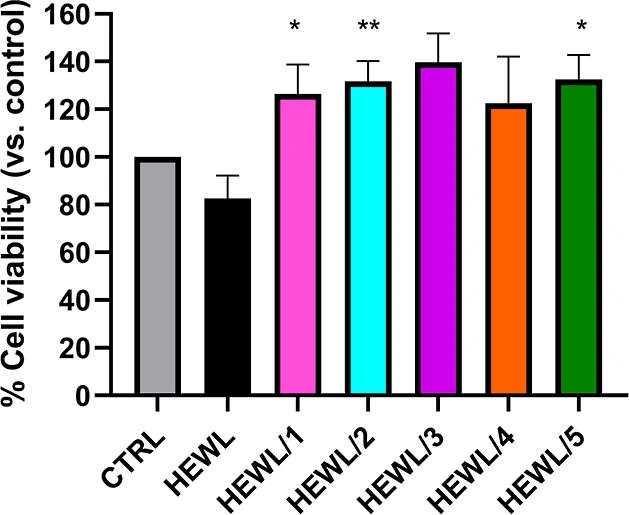
Effects of Ru_2_ complexes **1**–**5** on the cytotoxicity of preaggregated HEWL in SH-SY5Y neuroblastoma cells (inhibition experiment). HEWL (1.5 mM) was incubated either alone or in the presence of Ru_2_ complexes (1:1 HEWL/Ru_2_ complex molar ratio) in 20 mM Gly–HCl buffer (pH 2.0) at 50 °C for 7 days. The MTS assay was performed after 24 h of incubation with the HEWL samples (final protein concentration: 25 μM). Control cells (untreated) were considered as 100% viable. Cell viability is expressed as a percentage of control and reported as mean ± SD of three independent experiments. Statistical analysis was performed using one-way analysis of variance, followed by Bonferroni’s multiple comparisons test. *p ≤ 0.05; **p ≤ 0.01.

Overall, the data support a coordination-structure/activity relationship in which ligand steric, charge distribution, and axial-site accessibility collectively dictate both protein recognition and aggregate remodeling outcomes.

## Experimental Section

### Materials and Synthesis of Ru_2_ Compounds

HEWL was purchased from Sigma Chemical Co and used without further purification. The Ru_2_ complexes **1**–**5** were synthesized according to published procedures.^34,36^ The following is a summary of their syntheses.

### Synthesis of [Ru_2_Cl­(L–L)­(O_2_CCH_3_)_3_] (1–4)

These complexes were prepared according to the literature method as follows.^36^ A round-bottomed flask was charged with 50 mL of ethanol, 0.90 mmol of [Ru_2_Cl­(O_2_CCH_3_)_4_], and the appropriate *N*,*N′*-diarylformamidine derivative *N,N′*-bis­(4-cyanophenyl)­formamidine, compound **1**; *N,N′*-bis­(2-tolyl)­formamidine, compound **2**; *N,N′*-bis­(4-tolyl)­formamidine, compound **3**; or *N*-4-tolylamide, compound **4**, in a 1:1 molar ratio (0.90 mmol each). Subsequently, 300 μL triethylamine was introduced into the reaction vessel. The resulting heterogeneous mixture was subjected to ultrasonic irradiation at 80 kHz under ambient conditions for varying durations depending on the substrate: 180 min for **1**, 210 min for **2**, 150 min for **3**, and 300 min for **4**. Upon sonication, the reaction mixture was passed through diatomaceous earth to remove any insoluble particulates, and the filtrate was concentrated to dryness under reduced pressure. The resulting crude residue was washed with 50 mL of diethyl ether and subsequently dissolved in 150 mL of deionized water. This aqueous solution was again filtered through diatomaceous earth. The aqueous phase was transferred to a separatory funnel, treated with 5 mL of brine, and extracted twice with 50 mL of dichloromethane. The combined organic extracts were dried over anhydrous magnesium sulfate, filtered to remove the desiccant, and evaporated to dryness in vacuo. In the case of compound **4**, an additional purification step was implemented, wherein the solid was washed with 10 mL of hexane/acetone mixture (10:6 v/v). Finally, all products were subjected to prolonged drying under vacuum to afford the desired compounds.

### Synthesis of [Ru_2_(L–L)­(O_2_CCH_3_)_2_(O_2_CO)] (5)

This complex was prepared according to the literature method as follows.^34^ A round-bottomed flask was charged with 20 mL of ethanol and 0.10 mmol (66 mg) of [Ru_2_Cl­(D-*p*-FPhF)­(O_2_CCH_3_)_3_] (D-*p*-FPhF *= N,N′*-bis­(4-fluorophenyl)­formamidinate). To this stirring solution at ambient temperature, an aqueous solution of 0.10 mmol of K_2_CO_3_ (11 mg) dissolved in 0.3 mL of deionized water was added dropwise. The resulting mixture was allowed to react under magnetic agitation for a period of 5 h. Following this, 1 mL of 0.1 M AgNO_3_ aqueous solution was introduced into the reaction vessel, and the flask was carefully wrapped in aluminum foil to ensure complete protection from ambient light. Then, the suspension was filtered through diatomaceous earth to separate the precipitated AgCl byproduct. The filtrate was concentrated to dryness under reduced pressure. This crude solid was subsequently redissolved in 150 mL of distilled water and transferred to a separatory funnel. The aqueous phase was first washed with 50 mL of dichloromethane to eliminate any unreacted diruthenium starting material. Thereafter, the aqueous layer was subjected to five extractions with 100 mL of ethyl acetate. The combined organic extracts from the ethyl acetate phase were dried over anhydrous magnesium sulfate, filtered off, and concentrated to dryness under vacuum.

### HEWL Aggregation

For inhibition experiments, HEWL (1.5 mM) was incubated either alone or in the presence of Ru_2_ complexes (1:1 HEWL/Ru_2_ complex molar ratio) in 20 mM Gly–HCl buffer (pH = 2) in a water bath at 60 °C under constant stirring (700 rpm).

For disaggregation experiments, HEWL (1.5 mM) was first incubated alone under the same conditions to allow aggregate formation. Subsequently, Ru_2_ complexes were added to the preformed aggregates at a 1:1 molar ratio.

The aggregation process was monitored over time by withdrawing 25 μL aliquots at selected time points and storing them at 4 °C until analysis.

### UV–vis Absorption Spectroscopy

The solution stability of the metal complexes **1**–**5** at pH = 2 was evaluated by collecting UV–Vis absorption spectra in 20 mM Gly–HCl buffer (pH = 2) over 8 days. The compounds were dissolved in water (stock solutions, 2 mM) and then diluted in the buffer to reach a final concentration of 50 μM. UV–Vis spectra were collected using a BioDrop Duo UV–Visible spectrophotometer (Cambridge, United Kingdom) at room temperature, using 1 cm path length cuvettes and the following parameters: 290–600 nm range, 200 nm/min scan rate, and 2.0 nm bandwidth.

### Fluorescence Assays

Aggregation profiles were monitored using ThT fluorescence. Measurements were performed in black 96-well plates under continuous stirring using an EnVision 2105 fluorescence plate reader (PerkinElmer). Fluorescence measurements were collected at selected time points (Figure 4) with excitation and emission wavelengths set at 440 and 483 nm, respectively. Assays were carried out at 25 °C using HEWL at a final concentration of 50 μM in 20 mM Gly–HCl buffer (pH = 2), in the absence or presence of metal complexes 1–5 at a 1:1 HEWL/Ru_2_ complex molar ratio. ThT was added to a final concentration of 25 μM. All measurements were performed in duplicate.

### CD Spectroscopy

CD spectra of HEWL (50 μM) in 20 mM Gly–HCl buffer (pH = 2), in the absence and presence of metal complexes **1**–**5** at a 1:1 HEWL/Ru_2_ complex molar ratio, were recorded on a Jasco J-815 spectropolarimeter (JASCO, Tokyo, Japan) at 25 °C using a 0.1 cm path-length quartz cuvette.

### Dynamic Light Scattering

Measurements were performed using a Zetasizer Nano S instrument (Malvern Instruments, Malvern, Worcestershire, UK) equipped with a 633 nm laser operating at a backscatter detection angle of 173°. The instrument was thermostated using a Peltier system, and measurements were carried out in plastic microcuvettes. HEWL (50 μM) in 20 mM Gly–HCl buffer (pH = 2) at 25 °C was analyzed either alone or in the presence of metal complexes **1**–**5** at a 1:1 HEWL/Ru_2_ complex molar ratio under stirring. Size distributions by intensity were determined in automatic mode at regular time intervals over a period of 10 min for each measurement. Thirteen acquisitions were recorded, each with a duration of 10 s.

### Electrospray Ionization Mass Spectrometry

HEWL was dissolved either in water or in 20 mM Gly–HCl buffer (pH = 2) to a final concentration of 50 μM. The protein was incubated for 24 h with Ru_2_ complexes **1**–**5** at a 1:1 HEWL/Ru_2_ complex molar ratio, and each mixture was analyzed by native ESI–MS LTQ XL Ion Trap mass spectrometer equipped with an ESI source, operating at a needle voltage of 3.5 kV and temperature of 320 °C (Thermo Fisher Scientific). Spectra of the complexes alone were recorded as controls. Analyses were performed by direct injection at 10 μL·min^–1^. Spectra were acquired over an *m*/*z* range of 500–2000.

### SEM Analysis

The morphological features of HEWL aggregates, both in the absence and presence of Ru_2_ complexes **1**–**5**, were examined by field-emission SEM (FE-SEM; Phenom ProX, Thermo Fisher Scientific). Both inhibition and disaggregation assays of HEWL protein alone (50 μM) and incubated with Ru_2_ complexes at a 1:1 HEWL/Ru_2_ complex molar ratio in 20 mM Glycine-HCl buffer (pH = 2) were incubated for 7 days under aggregation-promoting conditions at 25 °C using a Multi Reax Vortexer 115 V (Heidolph Instruments GmbH & Co.). After incubation, aliquots (approximately 30 μL) were deposited onto aluminum stubs and left to dry under ambient conditions. The dried specimens were coated with a thin conductive gold layer by sputtering (25 mA, 75 s) using a LUXOR^Au^ SEM Coater (Aptco Technologies). Subsequently, samples were introduced into the microscope chamber, and images were recorded at an accelerating voltage ranging from 10 to 15 kV with a secondary electron detector.

### Crystallization, Data Collection, and Refinement of HEWL/1–5 Adducts

HEWL was purchased from Sigma Chemical Co (Merck Life Science S.r.l., Milan, Italy) at the highest grade of purity. To obtain HEWL/**1**–**5** adducts, first metal-free HEWL crystals were grown by hanging drop vapor diffusion in 20% ethylene glycol, 0.1 M sodium acetate at pH 4.0, and 0.6 M sodium nitrate. Then, crystals were soaked for 14 days using saturated solutions of the corresponding Ru_2_ compound (**1**–**5**) freshly dissolved in the reservoir. The HEWL/**1**–**5** crystals were flash-cooled in liquid N_2_ and stored under cryogenic conditions until data collection.

X-ray diffraction data were collected at the XRD2 beamline of the Elettra synchrotron in Trieste, Italy. Crystals were flash-frozen at 100 K using liquid nitrogen (and maintained at 100 K during the data collection). X-ray diffraction data were processed using autoPROC.^42^ The structure was solved by molecular replacement using the program Phaser of the CCP4 suite,^43^ and the model was derived from PDB code193L.^44^ The building of the model was carried out using Coot.^45^ The structure was refined using REFMAC5.^46^ Data collection and refinement statistics are summarized in Table S1. Coordinates and structure factors were deposited in the Protein Data Bank under the accession codes 29LJ (HEWL/**1**), 29MY (HEWL/**2**), 29NX (HEWL/**3**), 29MN (HEWL/**4**), and 29MR (HEWL/**5**).

### Cell Culture

SH-SY5Y human neuroblastoma cells (CRL-2266, ATCC, Manassas, VA, USA) were cultured in 100 × 20 mm dishes (1 × 10^6^ cells per dish) in DMEM (Corning; Product No. 10–013-CV) supplemented with 10% FBS (Sial; Product No. YourSIAL-FBS-SA), 100 U/mL penicillin, 100 μg/mL streptomycin (Corning; Product No. 30–002-CI), and 25 mM HEPES (Sigma-Aldrich; Product No. H0887). Cells were maintained in a humidified atmosphere containing 5% CO_2_ at 37 °C and were passaged upon reaching approximately 85% confluence.

### Cytotoxicity Assay (MTS Assay)

HEWL (1.5 mM) was incubated either alone or in the presence of Ru_2_ complexes (1:1 HEWL/Ru_2_ complex molar ratio) in 20 mM Gly–HCl buffer (pH = 2) for 7 days. Samples were then diluted in a cell culture medium at a final concentration of 25 μM and added to the cells for 24 h. Control cells were incubated with Gly–HCl buffer diluted in the cell culture medium at the same final concentration used for the protein samples.

Following treatment, cell viability was assessed using the MTS [3-(4,5-dimethylthiazol-2-yl)-5-(3-carboxymethoxyphenyl)-2-(4-sulfophenyl)-2H tetrazolium] assay (Cat. #G3580, CellTiter 96 Aqueous One Solution Cell Proliferation Assay, Promega, Milano, Italy). SH-SY5Y cells were seeded in 96-well plates at a density of 5.0 × 10^3^ cells per well and allowed to adhere prior to treatment. Following 24 h exposure, MTS reagent was added according to the manufacturer’s instructions, and absorbance was measured at 570 nm using a VICTOR Nivo microplate reader (Revvity). Background absorbance, determined from cell-free wells containing medium and MTS reagent, was subtracted from all readings. Cell viability was calculated by normalizing the mean absorbance values of treated cells to those of buffer-treated control cells and expressed as a percentage of control. In addition, the intrinsic cytotoxicity of the Ru_2_ complexes was evaluated independently under the same experimental conditions.

### Statistical Analysis

For cytotoxicity assays, statistical significance between groups was assessed by one-way analysis of variance followed by Bonferroni’s post hoc test for multiple comparisons, with *p* ≤ 0.05 considered significant. Analyses were performed using GraphPad Prism 8.0 (GraphPad Software, San Diego, CA, USA).

## Conclusions

In this study, we have systematically investigated the interaction between a series of five paddlewheel Ru_2_ complexes and HEWL, combining structural, spectroscopic, and biophysical approaches to elucidate their antiamyloid properties. X-ray crystallography demonstrates that all complexes bind preferentially to surface-exposed aspartate residues, primarily Asp119, through coordination to the Ru_2_ core, while preserving the native protein fold. The observation of both covalent and noncovalent binding modes (noncovalent interactions were previously unreported for cationic species) underscores the versatility of these metallodrugs and highlights the influence of steric hindrance of the equatorial ligands on the protein recognition properties of Ru_2_ compounds. Functional assays reveal that all Ru_2_ complexes effectively inhibit HEWL fibril formation, although their disaggregation efficiencies differ significantly. In particular, the complex with vacant axial coordination sites (**5**) exhibits enhanced ability to remodel preformed aggregates, suggesting a key role of axial ligand lability in modulating activity. Complementary CD, DLS, and SEM analyses indicate that these compounds do not fully revert fibrils to monomeric protein but instead promote partial disassembly and structural reorganization into less ordered aggregates. Furthermore, cytotoxicity assays demonstrated that Ru_2_ complexes significantly mitigate HEWL-induced toxicity in SH-SY5Y cells under inhibition conditions.

Collectively, our results demonstrate that paddlewheel diruthenium complexes behave as coordination-programmed modulators of amyloid assembly, in which subtle variations in ligand architecture and axial coordination environment translate into distinct protein-binding and aggregation-remodeling behaviors. The combined crystallographic and biophysical evidence establishes a direct link between Ru_2_ coordination chemistry and functional antiamyloid activity, providing a mechanistic framework for the rational development of multifunctional metallodrugs targeting protein misfolding processes.

## Supplementary Material



## Notes

Data collection and refinement statistics; UV–vis absorption spectra of the Ru_2_ complexes **1**–**5** at a final concentration of 50 μM in 20 mM Gly-HCl buffer, pH = 2; ESI-MS spectra of HEWL (50 μM) in the presence of compounds 2–5 at a 1:1 HEWL/Ru_2_ complex molar ratio, after 24 h of incubation. Buffer: 20 mM Gly-HCl buffer, pH = 2; experimental *m*/*z* values detected in the spectra of HEWL in the absence and in the presence of compound **1**, in water and in 20 mM Gly-HCl buffer at pH = 2; experimental *m*/*z* values detected in the spectra of HEWL alone and with the addition of Ru_2_ complexes **2**–**5**. Buffer: 20 mM Gly-HCl buffer, pH 2; overlay of CD spectra of HEWL (50 μM) in the absence and presence of Ru_2_ complexes **1**–**5** obtained from the disaggregation experiment, and hydrodynamic size parameters obtained by dynamic light scattering for preformed HEWL aggregates in the absence and presence of Ru_2_ complexes **1**–**5** (PDF)

## Abbreviations

AD: Alzheimer’s disease
APCF: Advanced Protein Crystallization Facility
ATCC: American Type Culture Collection
CD: circular dichroism
CCP4: Collaborative Computational Project No. 4
CO2: carbon dioxide
DLS: dynamic light scattering
DMEM: Dulbecco’s modified Eagle’s medium
ESI-MS: electrospray ionization mass spectrometry
FE-SEM: field-emission scanning electron microscopy
FBS: fetal bovine serum
Gly–HCl: glycine hydrochloride
HEWL: hen egg white lysozyme
HEPES: 4-(2-hydroxyethyl)-1-piperazineethanesulfonic acid
MTS: 3-(4,5-dimethylthiazol-2-yl)-5-(3-carboxymethoxyphenyl)-2-(4-sulfophenyl)-2H-tetrazolium
NCB: noncovalently bonded fragments
NRRP: National Recovery and Resilience Plan
PDB: Protein Data Bank
PDI: polydispersity index
REFMAC: reciprocal-space refinement of macromolecular structures
rmsd: root-mean-square deviation
Ru2: paddlewheel diruthenium complexes
SED: secondary electron detector
SEM: scanning electron microscopy
ThT: thioflavin T
UV–Vis: ultraviolet–visible spectroscopy.
