# Supplementary material for: Protein Recognition and Amyloid Remodeling Governed by Paddlewheel Diruthenium Coordination Chemistry
Source: Inorg Chem. 2026 Aug 19;65(34):19953–64. doi: 10.1021/acs.inorgchem.6c02947 (PMC13536882; doi:10.1021/acs.inorgchem.6c02947)
Supplement: Supplementary file 1 [file ic6c02947_si_001.pdf]

SUPPORTING INFORMATION

Protein Recognition and Amyloid Remodeling Governed by Paddlewheel Diruthenium Coordination Chemistry

Sara La Manna[a,], Daniele Florio[b], Giarita Ferraro[c], Maria Rosaria Policino[a], Filomena Altieri[b], Aarón Terán[d], Santiago Herrero[e,f], Antonello Merlino[c], Daniela Marasco[a,*]*

[a] Department of Pharmacy, University of Naples Federico II, 80131, Naples, Italy.

[b] IRCCS SYNLAB SDN, Via G. Ferraris 144, Naples, 80146, Italy.

[c] Department of Chemical Sciences, University of Naples Federico II, 80126, Naples, Italy.

[d] Structural Biology Programme, Spanish National Cancer Research Centre (CNIO), Melchor Fernández Almagro 3, 28029, Madrid, Spain.

[e] MatMoPol Research Group, Department of Inorganic Chemistry, Faculty of Chemical Science, Complutense University of Madrid, Avenida Complutense s/n, 28040, Madrid, Spain.

[f] Knowledge Technology Institute, Complutense University of Madrid, Campus de Somosaguas, 28223, Pozuelo de Alarcón, Madrid, Spain.

*Corresponding authors: Daniela Marasco, e-mail: daniela.marasco@unina.it; Sara La Manna, e-mail: sara.lamanna@unina.it

Table S1: Data collection and refinement statistics.

**Adduct**	HEWL/**1**	HEWL/**2**	HEWL/**3**	HEWL/**4**	HEWL/**5**
**PDB entry**	29LJ	29MY	29NX	29MN	29MR
**Crystallization conditions**	20 % ethylene glycol, 0.1 M sodium acetate at pH 4.0, and 0.6 M sodium nitrate				
**Data collection**					
**Space group**	$P42_12$	$P4_32_12$	$P4_12_12$	$P4_12_12$	$P4_12_12$
**a (Å)**	78.18	77.69	77.53	78.12	77.83
**b (Å)**	78.18	77.69	77.53	78.12	77.83
**c (Å)**	37.53	37.51	37.55	37.58	37.20
**α=β=γ (°)**	90.0	90.0	90.0	90.0	90.0
**Resolution range (Å)**	55.28-1.13 (1.15-1.13)	38.84-1.75 (1.77-1.75)	54.82-1.30 (1.32-1.30)	55.24-1.31 (1.33-1.31)	38.92-1.34 (1.36-1.34)
**Observations**	36587 (14141)	264324 (13576)	370221 (17706)	365776 (17908)	338863 (17114)
**Unique reflections**	32711 (1588)	12107 (614)	28691 (1400)	30924 (1403)	26206 (1288)
**Completeness (%)**	99.9 (100.0)	99.4 (100.0)	99.9 (100.0)	99.6 (100.0)	99.9 (100.0)
**Redundancy (Multiplicity)**	9.9 (8.9)	21.8 (22.1)	12.9 (12.6)	12.9 (12.8)	12.9 (13.3)
**†Rmerge (%)**	0.042 (0.836)	0.236 (1.025)	0.033 (1.111)	0.040 (1.187)	0.041 (1.184)
**Average I/σ(I)**	21.8 (2.2)	9.7 (3.7)	31.5 (2.2)	27.0 (2.1)	27.0 (2.2)
**CC1/2**	1.000 (0.800)	0.993 (0.805)	1.000 (0.775)	1.000 (0.806)	1.000 (0.808)
**Anom. completeness (%)**	99.9 (99.6)	99.4 (100.0)	99.9 (100.0)	99.8 (100.0)	99.9 (100.0)
**Anom. Multiplicity**	5.3 (4.7)	11.9 (11.8)	6.9 (6.6)	6.9 (6.7)	6.9 (7.0)
**Refinement**					
**Resolution (Å)**	1.13	1.75	1.30	1.31	1.34
**N° reflections**	36587	11491	27342	26987	20965
**N° reflections in working set**	1837	586	1364	1382	1167
R_{factor}/R_{free}	0.198/0.209	0.222/0.252	0.199/0.227	0.183/0.194	0.193/0.226
**N° non-H atoms in the refin.**	1266	1205	1354	1296	1270

**Ru_2 complex occupancy**	0.20 (NCB) 0.45 (bound to Asp119)	0.40 (bound to Asp101) 0.45 (bound to Asp119)	0.40 (NCB) 0.40 (bound to Asp119) 0.35 (NCB)	0.20 (bound to Asp101) 0.20 (bound to Asp119)	0.30 (bound to Asp101) 0.40 (bound to Asp119)
**Ramachandran statistics**					
**Most favoured**	131 (98 %)	132 (96 %)	131 (98 %)	140 (99 %)	136 (99 %)
**Outliers**	0	0	0	0	0
**Rmsd bonds (Å)**	0.012	0.007	0.011	0.011	0.010
**Rmsd angles (°)**	1.993	1.677	2.068	1.985	2.024

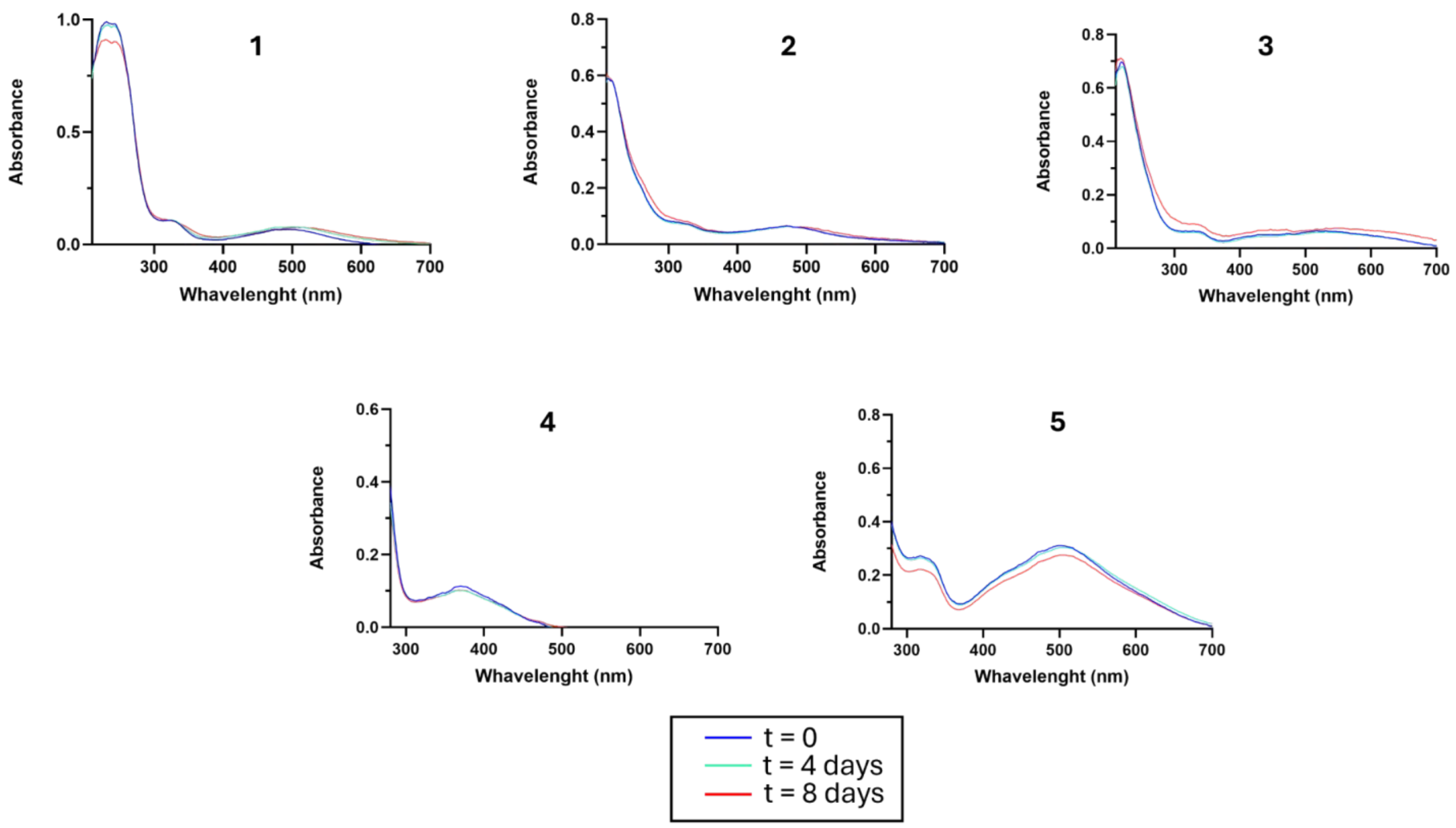

Figure S1. UV–vis absorption spectra of the Ru_2 complexes **1–5** at a final concentration of 50 µM in 20 mM Gly-HCl buffer, pH = 2.

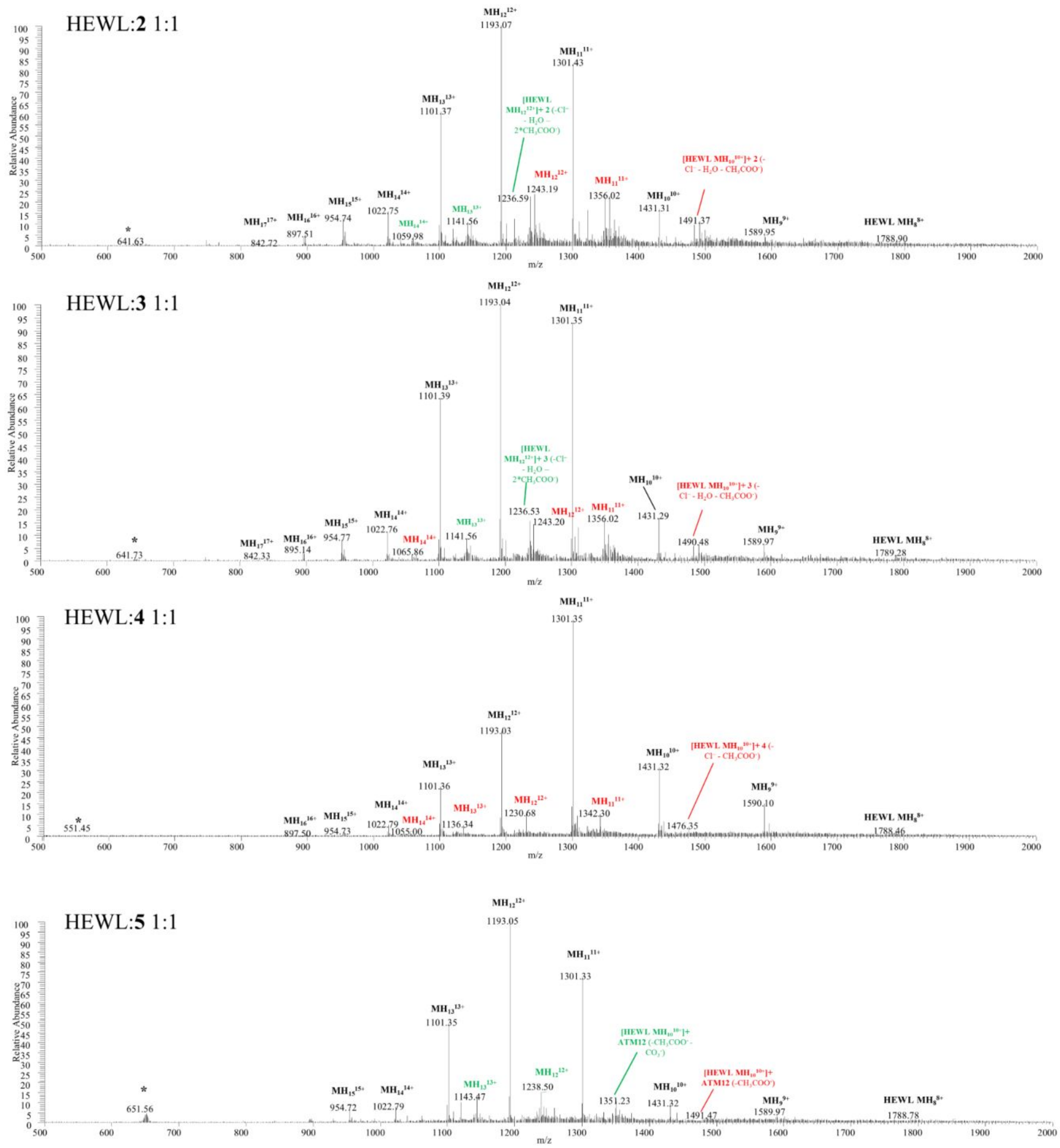

Figure S2. ESI-MS spectra of HEWL (50 µM) in presence of compounds **2–5** at a 1:1 HEWL/Ru_2 complex molar ratio, after 24 h of incubation. Buffer 20 mM Gly-HCl buffer, pH = 2. The asterisk highlights the species that are present also in the MS spectra of the complexes alone.

Table S2: Experimental *m/z* values detected in the spectra of HEWL in the absence and in the presence of compound **1**, in water and in 20 mM Gly-HCl buffer at pH = 2. The ion species corresponding to each experimental *m/z*, the expected *m/z* value (theoretical) and their charge states are also reported.

**Condition**	**Description**	***m/z* (charge)**	**Theoretical *m/z***
water	HEWL	1788.89 (+8)	1788.50
		1590.35 (+9)	1589.89
		1431.44 (+10)	1431.00
		1301.42 (+11)	1301.00
		1193.07 (+12)	1192.67
		1101.41 (+13)	1101.00
		1022.82 (+14)	1022.43
		956.14 (+15)	954.33
	HEWL + **1** (-Cl^-, -H_2O, -CH_3COO^-)	1859.00 (+8)	1859.19
		1653.05 (+9)	1652.73
		1487.86 (+10)	1487.56
		1352.63 (+11)	1352.41
		1240.02 (+12)	1239.80
		1144.70 (+13)	1144.50
		1063.05 (+14)	1062.83
	HEWL + 2·**1** (-2Cl^-, -2H_2O, -2CH_3COO^-)	1929.54 (+8)	1929.89
		1715.17 (+9)	1715.57
		1543.70 (+10)	1544.11
		1403.60 (+11)	1403.83
Gly-HCl buffer pH = 2	HEWL	1789.04 (+8)	1788.50
		1590.39 (+9)	1589.89
		1431.43 (+10)	1431.00
		1301.45 (+11)	1301.00
		1193.09 (+12)	1192.67
		1101.45 (+13)	1101.00
		1022.89 (+14)	1022.43
		954.85 (+15)	954.33
	HEWL + **1** (-Cl^-, -H_2O, -CH_3COO^-)	1859.58 (+8)	1859.19
		1652.71 (+9)	1652.73
		1487.80 (+10)	1487.56
		1352.63 (+11)	1352.41
		1239.92 (+12)	1239.80
		1144.88 (+13)	1144.50

Table S3: Experimental *m/z* values detected in the spectra of HEWL alone and with the addition of Ru_2 complexes **2–5.** Buffer 20 mM Gly-HCl buffer, pH 2. The ion species corresponding to each experimental *m/z*, the expected *m/z* value (theoretical) and their charge states are also reported.

	Description	*m/z* (charge)	Theoretical *m/z*
HEWL/**2**	HEWL	1788.9 (+8)	1788.50
		1589.95 (+9)	1589.89
		1431.31 (+10)	1431.00
		1301.43 (+11)	1301.00
		1193.07 (+12)	1192.67
		1101.37 (+13)	1101.00
		1022.75 (+14)	1022.43
		954.74 (+15)	954.33
		897.51 (+16)	849.75
		842.72 (+17)	842.18
	HEWL + **2** (-Cl^-, -H_2O, -CH_3COO^-)	1491.37 (+10)	1490.10
		1356.02 (+11)	1355.30
		1243.19 (+12)	1242.50
	HEWL + **2** (-Cl^-, -H_2O, -2·CH_3COO^-)	1236.59 (+12)	1236.59
		1141.56 (+13)	1141.54
		1059.98 (+14)	1060.07
HEWL/**3**	HEWL	1789.28 (+8)	1788.50
		1589.97 (+9)	1589.89
		1431.29 (+10)	1431.00
		1301.35 (+11)	1301.00
		1193.04 (+12)	1192.67
		1101.39 (+13)	1101.00
		1022.75 (+14)	1022.43
		954.74 (+15)	954.33
		897.51 (+16)	849.75
		842.72 (+17)	842.18
	HEWL + **3** (-Cl^-, -H_2O, -CH_3COO^-)	1490.48 (+10)	1490.10
		1356.02 (+11)	1355.30
		1243.20 (+12)	1242.50
		1065.86 (+14)	1064.29
	HEWL + **3** (-Cl^-, -H_2O, -2·CH_3COO^-)	1236.53 (+12)	1236.59
		1141.56 (+13)	1141.54
HEWL/**4**	HEWL	1788.46 (+8)	1788.50
		1590.10 (+9)	1589.89
		1431.32 (+10)	1431.00
		1301.35 (+11)	1301.00
		1193.03 (+12)	1192.67
		1101.36 (+13)	1101.00
		1022.79 (+14)	1022.43
		954.73 (+15)	954.33
		897.50 (+16)	849.75
	HEWL + **4** (-Cl^-, -CH_3COO^-)	1476.35 (+10)	1476.44

		1342.30 (+11)<br>1230.68 (+12)<br>1136.34 (+13)<br>1055.00 (+14)	1342.31<br>1230.54<br>1135.96<br>1054.89
HEWL/**5**	HEWL	1788.78 (+8)<br>1589.97 (+9)<br>1431.32 (+10)<br>1301.33 (+11)<br>1193.05 (+12)<br>1101.35 (+13)<br>1022.79 (+14)<br>954.72 (+15)	1788.50<br>1589.89<br>1431.00<br>1301.00<br>1192.67<br>1101.00<br>1022.43<br>954.33
	HEWL + **5** (-CH_3COO^-) or HEWL + **5** (-CO_3^-)	1491.47 (+10)	1491.65
	HEWL + **5** (- CH_3COO, -CO_3^-)<br>or<br>HEWL + **5** (-2·CH_3COO^-)	1351.23 (+11)<br>1238.50 (+12)<br>1143.47 (+13)	1350.80<br>1238.29<br>1143.12

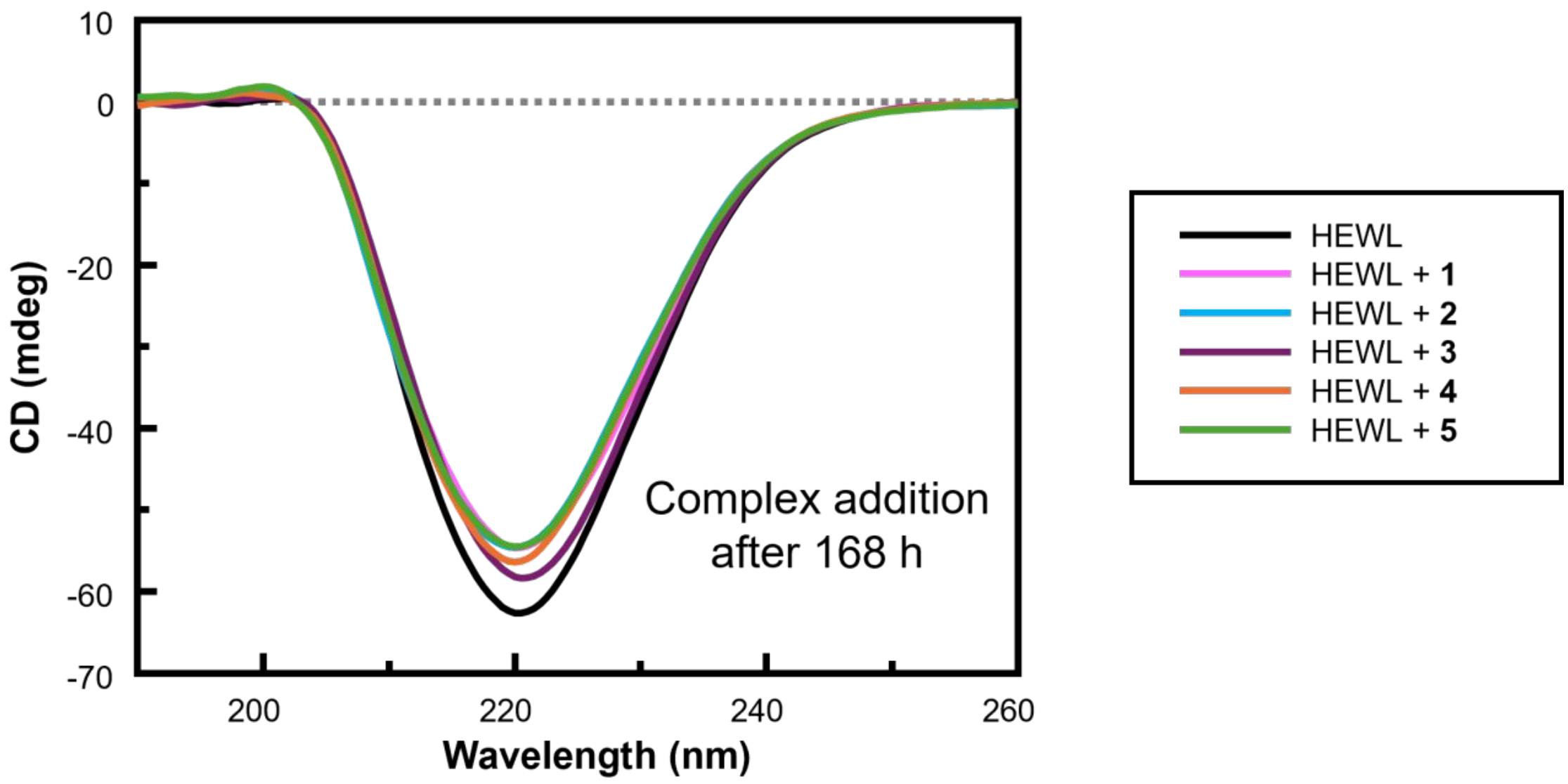

Figure S3. Overlay of CD spectra of HEWL (50 μM) in the absence and presence of Ru_2 complexes **1**–**5** obtained from the disaggregation experiment. Samples were prepared in 20 mM Gly–HCl buffer (pH = 2) at a 1:1 HEWL/Ru_2 complex molar ratio. Metal complexes were added after 168 h of HEWL incubation under aggregation-promoting conditions.

Table S4: Hydrodynamic size parameters obtained by dynamic light scattering for preformed HEWL aggregates in the absence and presence of Ru_2 complexes **1**–**5**.

Sample	Z-average (d/nm)	PDI	Size (d/nm)	St Dev (d/nm)
HEWL	219.4	0.337	336.6<br>44.71	199.3<br>9.890
HEWL/**1**	158.8	0.269	188.5	93.06
HEWL/**2**	166.0	0.336	211.7	127.5
HEWL/**3**	163.4	0.327	203.6	107.1
HEWL/**4**	169.5	0.336	212.9	95.03
HEWL/**5**	151.9	0.258	196.8	115.6

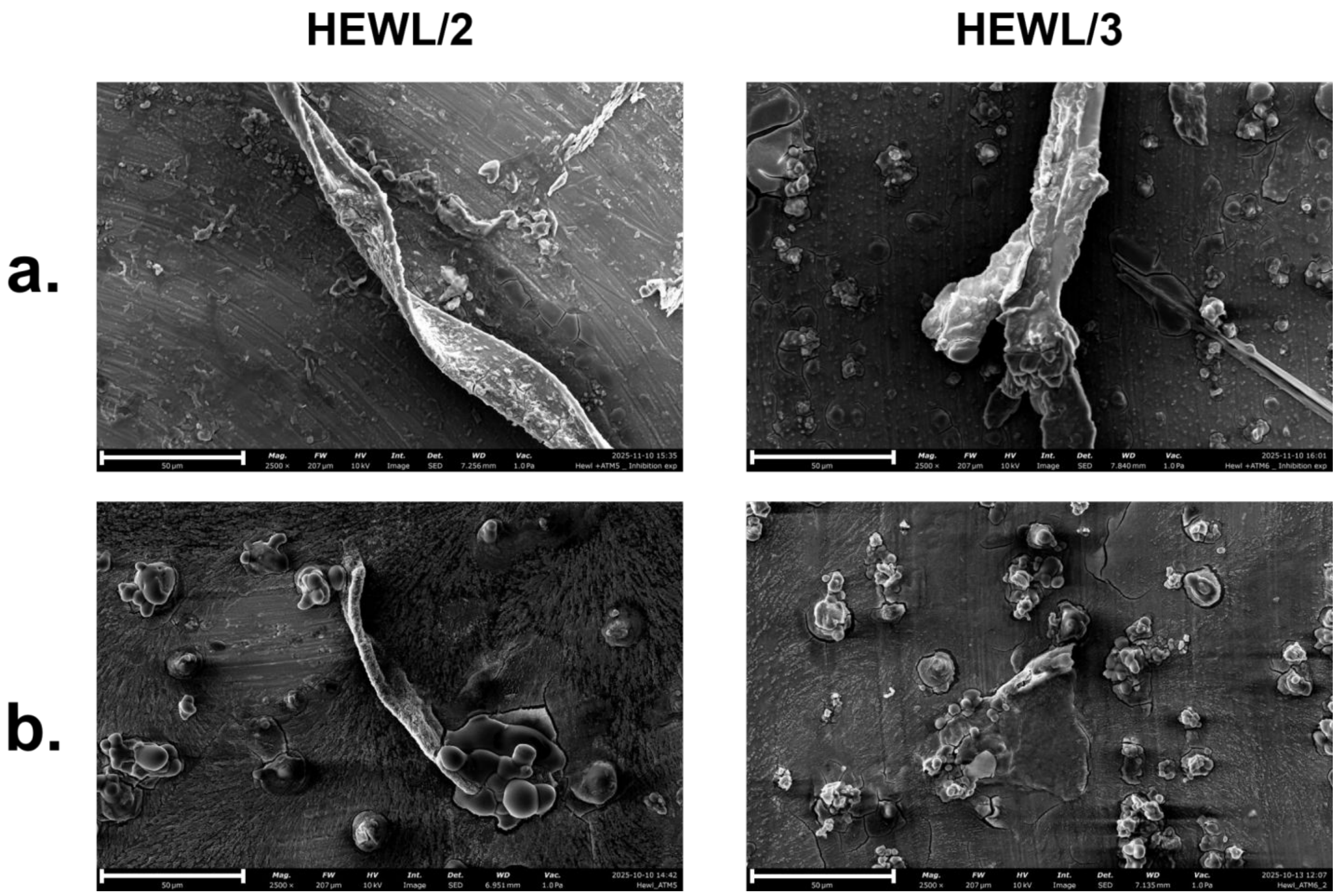

Figure S4. SEM of HEWL incubated alone and in the presence of **2** and **3** complexes, showing representative morphological features from inhibition (a) and disaggregation (b) experiments.

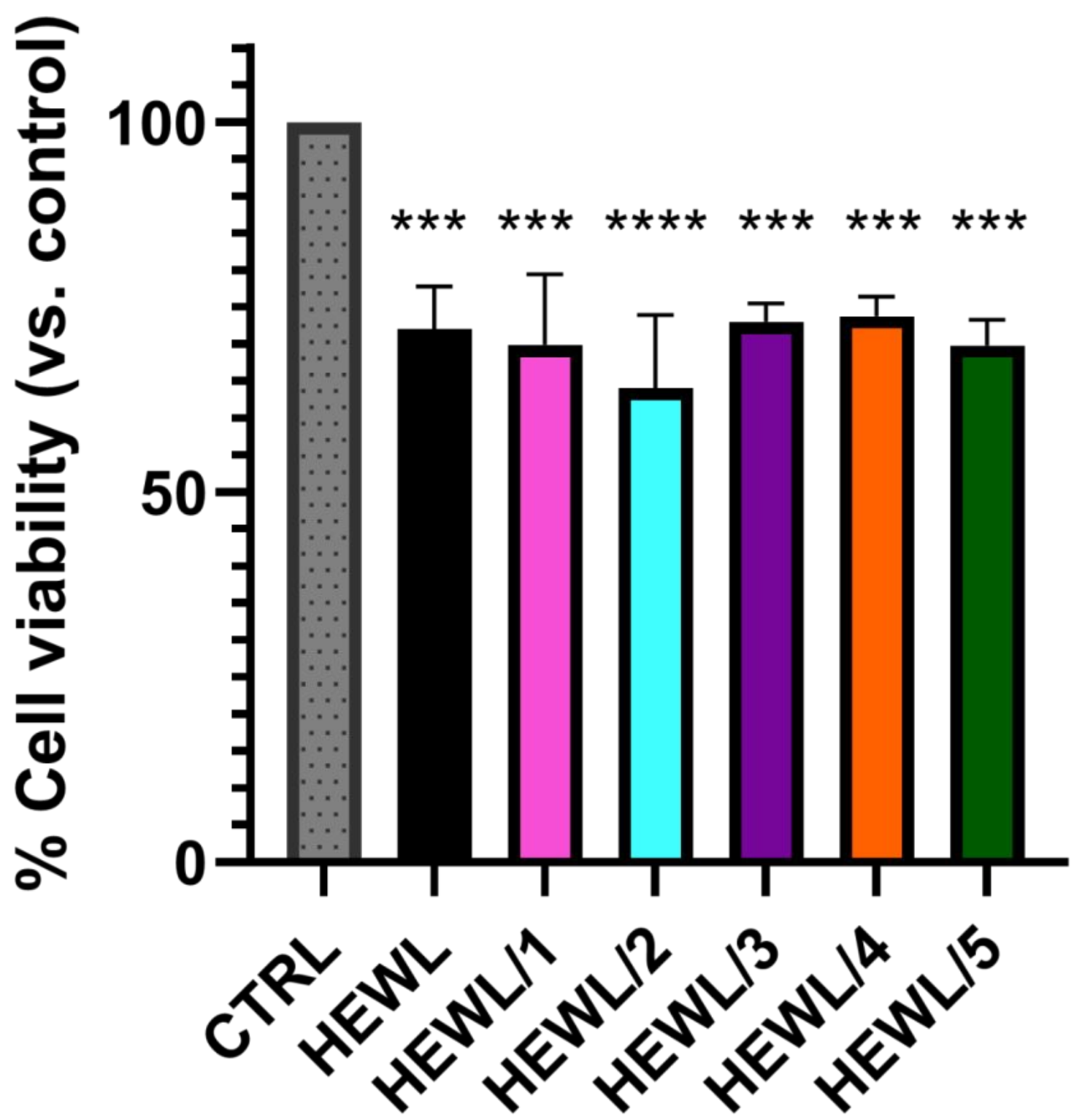

Figure S5. Effects of Ru_2 complexes **1**–**5** on the cytotoxicity of pre-aggregated HEWL in SH-SY5Y neuroblastoma cells (disaggregation experiment). MTS assay was performed after 24 h of incubation in the absence and presence of Ru_2 complexes **1**–**5**. Control cells (untreated) were considered as 100 % viable. Cell viability is expressed as percentage of control and reported as mean ± SD of three independent experiments. Statistical analysis was performed using one-way ANOVA followed by Bonferroni's multiple comparisons test. ***$p \leq 0.005$; ****$p \leq 0.001$.
